# Long-term cumulative exposure and change patterns of CTI-FI predict the risk of cardiometabolic multimorbidity in middle-aged and older Chinese adults

**DOI:** 10.1007/s40200-026-02026-y

**Published:** 2026-07-24

**Authors:** Chun-juan Zhang, Ling Zeng

**Affiliations:** Haiyan People’s Hospital, Affiliated Haiyan Hospital of Jiaxing University, Jiaxing, Zhejiang 314300 People’s Republic of China

**Keywords:** Cardiometabolic multimorbidity, C-reactive protein, Triglyceride-glucose index, Frailty index, CHARLS

## Abstract

**Background:**

Cardiometabolic multimorbidity (CMM) represents an increasing public health concern among aging populations. We aimed to construct and evaluate a multidimensional index integrating inflammation, metabolism, and physiological function–the CTI-FI (C-reactive protein–triglyceride-glucose and frailty index), and examine its association with incident CMM among middle-aged and older Chinese adults.

**Methods:**

Data were obtained from the China Health and Retirement Longitudinal Study (CHARLS), a nationally representative prospective cohort. Baseline CTI-FI and cumulative CTI-FI exposure (cuCTI-FI) were calculated, and CTI-FI change patterns were identified using K-means clustering based on repeated measurements. Cox proportional hazards models and restricted cubic spline analyses were performed to evaluate the associations between CTI-FI measures and incident CMM. The incremental value of cuCTI-FI beyond the China-PAR model was assessed using discrimination, calibration-related metrics, and decision curve analysis.

**Results:**

Among 4,438 participants, higher baseline CTI-FI and cuCTI-FI levels were consistently associated with increased risk of incident CMM in a dose–response manner (both P_trend < 0.001). In fully adjusted models, participants in the highest tertiles of baseline CTI-FI and cuCTI-FI had higher risks of incident CMM (HR: 3.47, 95% CI: 2.62–4.59; and HR: 5.27, 95% CI: 3.92–7.09, respectively). CTI-FI change pattern analysis suggested that participants with persistently higher or increasing CTI-FI levels had greater CMM risk compared with those with stable-low levels (HR: 4.89, 95% CI: 3.78–6.32). Incorporating cuCTI-FI into the China-PAR model improved model discrimination, with AUC increasing from 0.704 to 0.759. The associations remained generally consistent across most subgroup analyses.

**Conclusions:**

Higher baseline and cumulative CTI-FI levels were associated with increased risk of incident CMM among middle-aged and older Chinese adults. The addition of cuCTI-FI to conventional risk factors provided additional information for CMM risk assessment. Further validation in independent populations is needed to determine its potential application in broader risk assessment settings.

Clinical trial number.

Not applicable.

**Graphical abstract:**

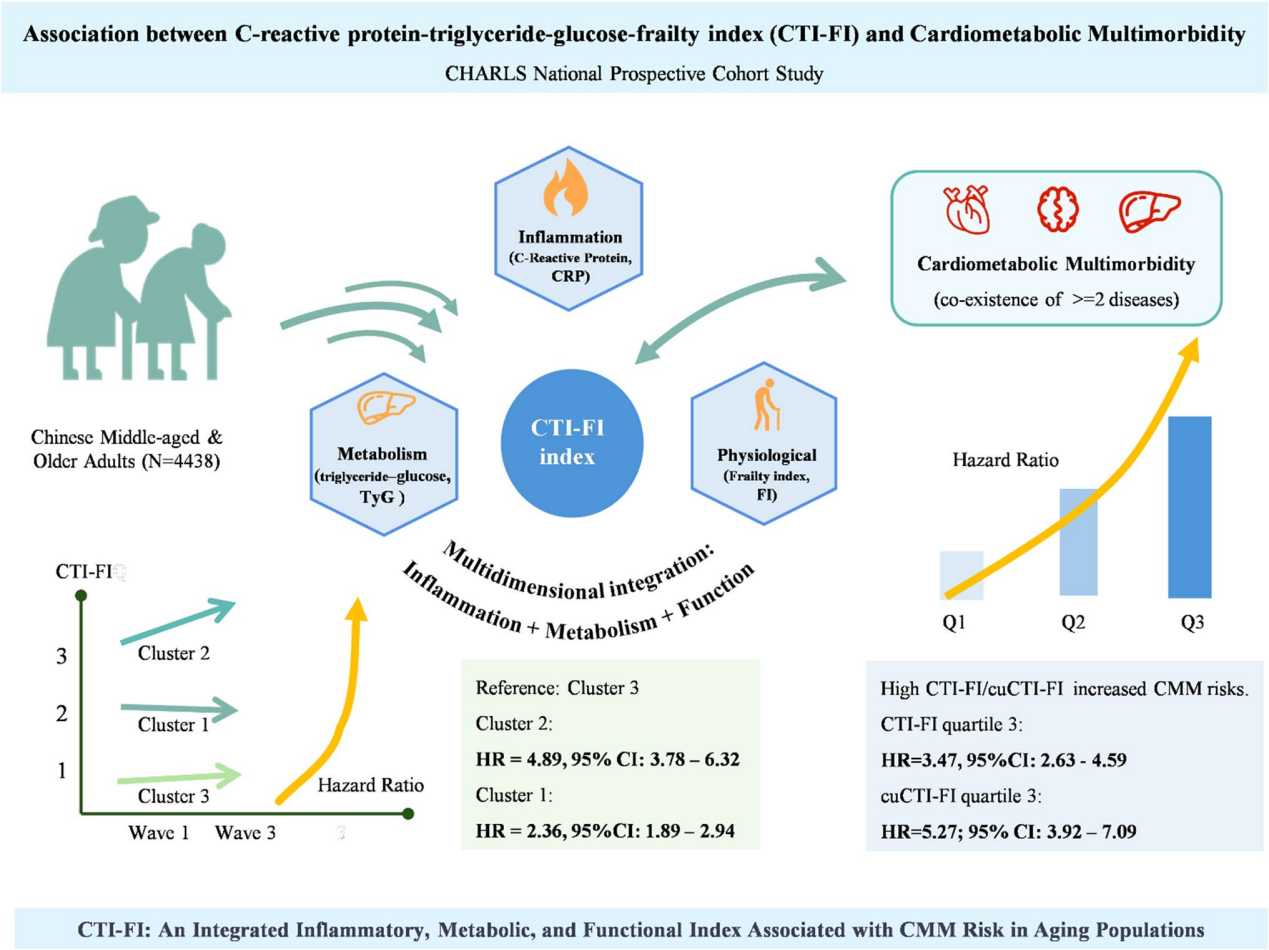

**Supplementary Information:**

The online version contains supplementary material available at 10.1007/s40200-026-02026-y.

## Introduction

Cardiometabolic multimorbidity (CMM), defined as the co-occurrence of at least two cardiometabolic diseases such as coronary heart disease, stroke, and type 2 diabetes, has emerged as a critical global public health challenge [1, 2]. In the context of an aging population and the escalating prevalence of obesity [3], the burden of CMM in China has reached 16.9% [4] and continues to rise. Compared with individuals having a single cardiometabolic condition, those with CMM experience significantly higher risks of all-cause mortality [5], cognitive decline [6], and depression[7], placing an immense strain on healthcare systems [8]. Despite this, current risk assessment for CMM remains suboptimal [9], largely relying on conventional clinical markers (e.g., blood pressure and lipid profiles) that often fail to capture the synergistic, multi-dimensional pathological processes underlying the progression of multimorbidity.

The pathogenesis of CMM is characterized by a complex interplay between metabolic dysregulation [10], chronic inflammation, and physiological decline [11]. Insulin resistance (IR) serves as a core driver, promoting oxidative stress and endothelial dysfunction through lipotoxicity and glucotoxicity. The triglyceride–glucose (TyG) index has recently gained attention as a reliable, cost-effective surrogate for IR and is independently associated with CMM risk [12]. Parallel to metabolic factors, chronic low-grade inflammation—typically measured by C-reactive protein (CRP)—amplifies atherothrombotic risk via immune-metabolic pathways [13]. Furthermore, frailty reflects a state of diminished physiological reserve and increased vulnerability to stressors, which can exacerbate the deleterious effects of both IR and inflammation [14, 15]. While emerging composite metrics, such as TyG-WHtR [16, 17],TyG combined with the Frailty Index (TyGFI) [18] and CTI (CRP-TyG), have demonstrated improved predictive value over single indicators [19, 20], they typically focus on isolated cardiovascular outcomes and often overlook the functional dimension of aging.

To date, several composite indicators have been developed to capture different aspects of cardiometabolic risk [21, 22]. However, few have simultaneously incorporated inflammatory status, metabolic dysfunction, and physiological vulnerability. To address this gap, we constructed the CTI-FI, a composite index integrating the CRP-TyG index (CTI) and the Frailty Index (FI), to capture multiple domains associated with cardiometabolic vulnerability. Using data from the China Health and Retirement Longitudinal Study (CHARLS), a large-scale prospective cohort of middle-aged and older Chinese adults, this study aimed to: (1) examine the association between CTI-FI and incident CMM; (2) evaluate dose–response relationships and CTI-FI change patterns over time; and (3) assess whether CTI-FI could provide additional information beyond conventional risk factors for CMM risk assessment. Our findings may contribute to a better understanding of the relationship between inflammatory, metabolic, and functional factors and CMM development.

## Methods

### Study design and population

This study used data from the CHARLS, a nationwide prospective cohort. The national baseline survey (Wave 1) was conducted between June 2011 and March 2012, enrolling 17,705 participants across 28 provinces. Follow-up surveys were conducted biennially through 2020. Detailed methodology of CHARLS has been described previously [23]. Ethical approval was obtained from the Peking University Biomedical Ethics Review Committee (IRB00001052-11015), and all participants provided written informed consent.

**Fig. 1:**
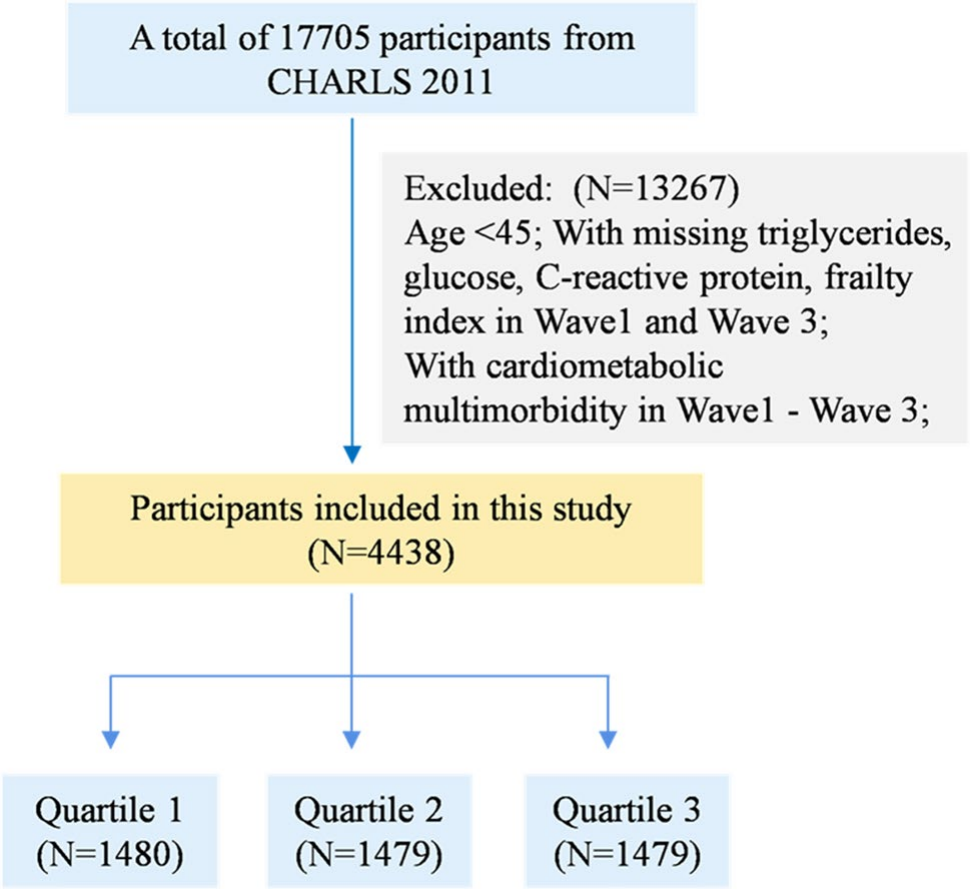
Flow chart of the study population

Initially, 17,705 participants were identified. We excluded those with missing baseline or Wave 3 data for triglycerides (TG), fasting blood glucose (FBG), C-reactive protein (CRP), or frailty index (FI). Participants aged < 45 years or those with prevalent CMM before Wave 3 were also excluded. Ultimately, 4,438 participants were included in the final analysis (Fig. 1). Wave 3 was considered as the landmark time point. Participants with prevalent CMM before Wave 3 were excluded. Follow-up started from Wave 3 and continued until the occurrence of incident CMM, loss to follow-up, or the last available follow-up wave. During 38,150 person-years of follow-up, 567 participants developed incident CMM, corresponding to an incidence rate of 14.86 per 1,000 person-years.

### Assessment of CTI, FI, CTI-FI, cuCTI-FI and CTI-FI changes

The TyG index, CTI were calculated via the following formulas: TyG index = ln [triglycerides (mg/dL) × glucose (mg/dL)/2]; CTI = 0.412 × ln(CRP [mg/L]) + ln(TG [mg/dL] × FPG [mg/dL])/2 [24].

The FI was constructed based on 29 items (including chronic diseases and functional impairments)[25, 26]. Each item was scored as 0 (absence of deficit) or 1 (presence of deficit). The FI was calculated as the sum of deficit scores divided by the total items (range 0–1), with higher scores indicating increased frailty [27, 28].

The composite CTI-FI was defined as CTI*FI. To evaluate long-term exposure, cumulative indices (cuTyG, cuCTI, cuFI, and cuCTI-FI) were calculated using a time-weighted average approach:

$$cuCTI=\frac{{CTI}_{2012}+{CTI}_{2015}}{2}\times (2015-2012)$$

where $\Delta \mathrm{t}$ represents the time interval (3 years) between surveys. This approach adheres to the methodology for cumulative metabolic indices in the CHARLS cohorts, which is consistent with the approach described by Zou [29] and Lu [30].

### Assessment of CMM

The primary outcome was incident CMM, which defined as the first occurrence of two or more cardiometabolic diseases (heart disease, stroke, and diabetes) during follow-up among participants who were free of CMM at the landmark point. Heart disease and stroke were identified via self-reported physician diagnosis or medication use. Diabetes was defined as: (1) self-reported physician diagnosis; (2) use of antidiabetic medication; (3) FBG ≥ 126 mg/dL (7.0 mmol/L); or (4) HbA1c ≥ 6.5% [29].

### Covariates

Covariates were categorized as: Demographics: Age, sex, marital status, education, and residence (rural/urban); Lifestyle: Smoking status and alcohol consumption; Medical History: Hypertension and cancer; Physical/Laboratory Measures: Blood pressure (SBP/DBP), lipid profiles (TC, HDL-C, LDL-C), and FBG; Hypertension was defined as SBP ≥ 140 mmHg, DBP ≥ 90 mmHg, self-reported history, or current antihypertensive therapy.

### Data preprocessing

Missing data were handled using Multiple Imputation by Chained Equations (MICE) (Table S1). Multicollinearity was assessed using the generalized variance inflation factor (GVIF), with (GVIF)^(1/2DF) < 2 considered acceptable (Table S2).

### Statistical analysis

Statistical analyses were performed using R software (version 4.3.3). Continuous variables are presented as mean ± standard deviation (SD) or median (interquartile range [IQR]), and categorical variables are presented as numbers and percentages. Participants were categorized into tertiles (Q1–Q3) according to baseline CTI-FI and cumulative CTI-FI (cuCTI-FI). Kaplan–Meier curves were generated to compare CMM-free survival among CTI-FI categories. Cox proportional hazards regression models were used to estimate hazard ratios (HRs) and 95% confidence intervals (CIs). Three sequential models were constructed: Model 1 adjusted for age and sex; Model 2 additionally adjusted for sociodemographic and lifestyle factors, including education, marital status, smoking, alcohol consumption, and body mass index; Model 3 further adjusted for baseline clinical characteristics, including comorbidities and HDL. Variables directly included in the calculation of CTI-FI (e.g., triglycerides and glucose-related indicators) were not additionally adjusted to avoid potential overadjustment. The proportional hazards assumption was evaluated using Schoenfeld residual tests, and no substantial violations were observed (continuous cuCTI-FI: P = 0.415, Global P = 0.294; categorical cuCTI-FI: P = 0.426, Global P = 0.360). Restricted cubic spline (RCS) analyses were performed to assess potential nonlinear dose–response associations between CTI-FI measures and incident CMM.

Several sensitivity analyses were conducted to evaluate the robustness of the findings, including: (1) complete-case analyses excluding participants with missing covariate data; (2) analyses excluding participants with baseline hypertension or cancer; (3) analyses excluding participants from the 2020 follow-up wave; and (4) competing risk analyses using Fine-Gray hazard models, considering death as a competing event. A two-sided P value < 0.05 was considered statistically significant.

## Results

### Baseline characteristics of participants

A total of 4,438 participants were included and categorized into tertiles based on baseline CTI-FI levels. As shown in Table 1, individuals in the highest tertile (Q3) were generally older, more likely to be female, had lower educational attainment, and predominantly resided in rural areas. Compared with the lower tertiles, participants in Q3 exhibited a higher prevalence of hypertension and heart disease. The proportion of participants developing incident CMM during follow-up was higher among individuals with higher CTI-FI levels. Additionally, anthropometric and metabolic profiles—including waist circumference, fasting blood glucose, and triglycerides—were significantly more adverse in the Q3 group. Baseline characteristics stratified by cuCTI-FI tertiles are provided in the Supplementary Material (Table S3).

**Table 1:** Baseline characteristics according to baseline CTI-FI tertiles

	Level	Overall	Q1	Q2 (N = 1479)	Q3	*P* value
		(N = 4438)	(N = 1480)		(N = 1479)	
Age (median [IQR])		59.0[53.0,65.0]	56.0[50.0,62.0]	58.0[53.0,64.0]	61.0[55.0,68.0]	< 0.001
Gender (%)	Female	2410(54.3)	668(45.1)	785(53.1)	957(64.7)	< 0.001
	Male	2028(45.7)	812(54.9)	694(46.9)	522(35.3)	
Education (%)	High education	1241(28.0)	590(39.9)	415(28.1)	236(16.0)	< 0.001
	Low education	3197(72.0)	890(60.1)	1064(71.9)	1243(84.0)	
Married (%)	married	3937(88.7)	1374(92.8)	1314(88.8)	1249(84.4)	< 0.001
	unmarried	501(11.3)	106(7.2)	165(11.2)	230(15.6)	
Location (%)	city	249(5.6)	107(7.2)	92(6.2)	50(3.4)	< 0.001
	village	4189(94.4)	1373(92.8)	1387(93.8)	1429(96.6)	
Drink (%)	No	2987(67.3)	897(60.6)	980(66.3)	1110(75.1)	< 0.001
	Yes	1451(32.7)	583(39.4)	499(33.7)	369(24.9)	
Smoking (%)	Ex-smoker	365(8.2)	105(7.1)	134(9.1)	126(8.5)	< 0.001
	Non-smoker	2741(61.8)	850(57.4)	894(60.4)	997(67.4)	
	Smoker	1332(30.0)	525(35.5)	451(30.5)	356(24.1)	
Hypertension (%)	No	2688(61.0)	1080(73.5)	881(60.0)	727(49.4)	< 0.001
	Yes	1721(39.0)	390(26.5)	587(40.0)	744(50.6)	
Heart problem (%)	No	4006(90.3)	1415(95.6)	1361(92.0)	1230(83.2)	< 0.001
	Yes	432(9.7)	65(4.4)	118(8.0)	249(16.8)	
Diabetes (%)	No	4166(94.3)	1435(97.3)	1382(94.1)	1349(91.5)	< 0.001
	Yes	252(5.7)	40(2.7)	87(5.9)	125(8.5)	
Lung disease (%)	No	3971(89.9)	1455(98.5)	1323(90.1)	1193(81.1)	< 0.001
	Yes	446(10.1)	22(1.5)	146(9.9)	278(18.9)	
Cancer (%)	No	4372(99.1)	1473(99.8)	1457(99.2)	1442(98.4)	< 0.001
	Yes	39(0.9)	3(0.2)	12(0.8)	24(1.6)	
Liver disease (%)	No	4223(96.0)	1442(97.8)	1408(96.2)	1373(93.9)	< 0.001
	Yes	176(4.0)	32(2.2)	55(3.8)	89(6.1)	
Stroke (%)	No	4368(98.9)	1475(99.9)	1461(99.3)	1432(97.5)	< 0.001
	Yes	50(1.1)	2(0.1)	11(0.7)	37(2.5)	
Kidney disease (%)	No	4140(94.0)	1423(96.5)	1387(94.5)	1330(91.1)	< 0.001
	Yes	263(6.0)	52(3.5)	81(5.5)	130(8.9)	
Stomach disease (%)	No	3388(76.7)	1280(86.6)	1127(76.5)	981(66.8)	< 0.001
	Yes	1032(23.3)	198(13.4)	347(23.5)	487(33.2)	
Emotional disease (%)	No	4379(98.7)	1472(99.5)	1468(99.3)	1439(97.3)	< 0.001
	Yes	59(1.3)	8(0.5)	11(0.7)	40(2.7)	
Memory disease (%)	No	4396(99.1)	1473(99.5)	1468(99.3)	1455(98.4)	0.003
	Yes	42(0.9)	7(0.5)	11(0.7)	24(1.6)	
Arthritis (%)	No	2896(65.3)	1344(90.8)	964(65.2)	588(39.8)	< 0.001
	Yes	1542(34.7)	136(9.2)	515(34.8)	891(60.2)	
Asthma (%)	No	4253(96.3)	1458(99.0)	1416(96.1)	1379(93.6)	< 0.001
	Yes	165(3.7)	14(1.0)	57(3.9)	94(6.4)	
Incident CMM during follow-up (%)	No	3871(87.2)	1397(94.4)	1309(88.5)	1165(78.8)	< 0.001
	Yes	567(12.8)	83(5.6)	170(11.5)	314(21.2)	
BMI (median [IQR])		23.1	23	23.2	23.2	0.102
		[20.9,25.7]	[20.9,25.3]	[21.0,25.8]	[20.7,26.1]	
Waist (median [IQR])		84.5	83.7	84.3	86	< 0.001
		[78.0,91.8]	[77.6,90.2]	[77.6,91.4]	[78.5,93.0]	
SBP (median [IQR])		126.7	124.3	126.0 [114.0,141.7]	129.5 [115.7,145.7]	< 0.001
		[114.3,141.3]	[113.7,137.7]			
DBP (median [IQR])		74.7 [67.0,83.0]	74	74.3	75.7	0.081
			[67.0,82.3]	[67.0,83.3]	[67.7,83.7]	
Glucose (median [IQR])		5.7[5.2,6.2]	5.6[5.2,6.1]	5.7	5.7	< 0.001
				[5.2,6.2]	[5.3,6.3]	
TG (median [IQR])		1.1[0.8,1.6]	1.1[0.8,1.5]	1.1[0.8,1.6]	1.2[0.9,1.8]	< 0.001
TC (median [IQR])		4.9[4.4,5.6]	4.9[4.3,5.5]	5.0[4.4,5.6]	5.0[4.4,5.6]	0.022
HDL (median [IQR])		1.3[1.1,1.6]	1.3[1.1,1.6]	1.3[1.1,1.6]	1.3[1.0,1.5]	0.036
LDL (median [IQR])		3.0[2.4,3.5]	3.0[2.4,3.5]	3.0[2.5,3.5]	3.0[2.4,3.5]	0.562

### Associations among CTI-FI, cuCTI-FI, CTI-FI change patterns, and incident CMM risk

**Fig. 2:**
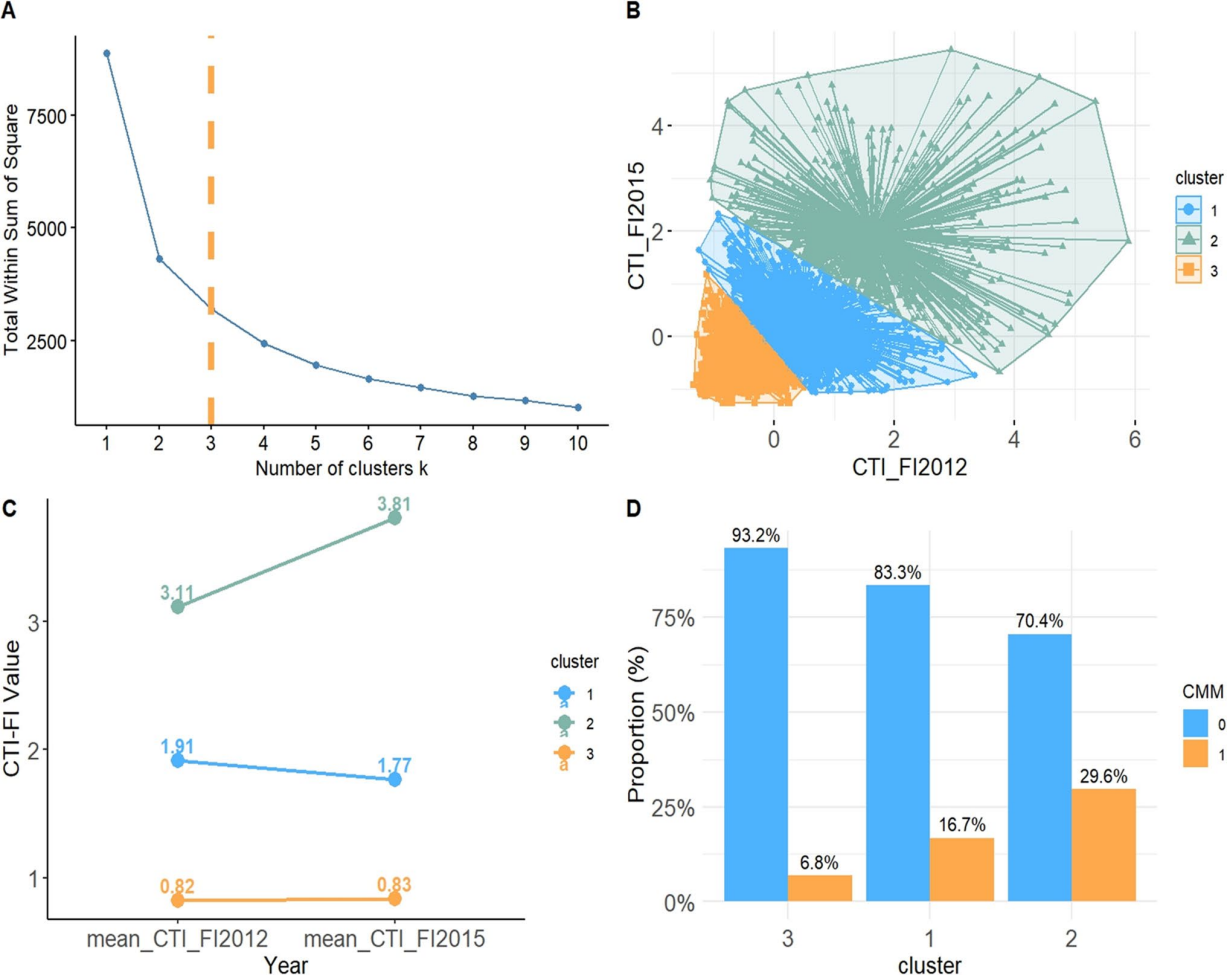
Clustering of the change in the CTI-FI from Wave 1 to Wave 3. (**A**) The elbow plot; (**B**) Three clusters were found via the K-means method with Euclidean distance; (**C**) Change in the CTI from wave 1 to wave 3; (**D**) Onset of CMM in three clusters

Based on standardized CTI-FI values measured at Wave 1 and Wave 3, K-means clustering identified three distinct longitudinal change patterns of CTI-FI (Fig. 2). These patterns were characterized as low-stable (Cluster 3), moderate (Cluster 1), and high-increasing (Cluster 2). The largest proportion of participants belonged to Cluster 3, whereas Cluster 2 showed the highest incidence proportion of CMM (29.6%). The number of clusters was evaluated using multiple criteria, including the elbow method, silhouette analysis, and Gap statistic. Bootstrap stability analysis showed high cluster reproducibility, with a mean Jaccard similarity coefficient of 0.933 across clusters, supporting the stability of the identified patterns (Figure S1).

Cox proportional hazards models revealed that higher CTI-FI, cuCTI-FI, and their respective change patterns were all positively associated with incident CMM, with significant dose–response trends (all P_trend < 0.001; Table 2). In the fully adjusted models, each one-unit increase in CTI-FI and cuCTI-FI was associated with a 40% (HR = 1.40) and 10% (HR = 1.10) increased risk of CMM, respectively. Compared with the lowest tertile (Q1), participants in the highest CTI-FI tertile had a 3.47-fold risk (HR = 3.47), while those in the highest cuCTI-FI tertile showed a 5.27-fold risk (HR = 5.27). Furthermore, relative to the low-stable (Cluster 3), the risk of CMM increased by 236% in Cluster 1 (HR = 2.36) and 489% in Cluster 2 (HR = 4.89).

**Table 2:** Multivariate Cox regression of the relationships between CTI-FI, cuCTI-FI, different clusters of CTI-FI and the risk of new-onset CMM

	Model 1		Model 2			Model 3	
	HR (95%CI)	*P*_*adj*_	HR (95%CI)	*P*_*adj*_		HR (95%CI)	*P*_*adj*_
CTI-FI	1.52 (1.43–1.61)	< 0.001	1.52 (1.43–1.63)	< 0.001		1.40 (1.29–1.51)	< 0.001
CTI-FI3							
Q1	Ref		Ref			Ref	
Q2	2.12 (1.63–2.75)	< 0.001	2.16 (1.66–2.82)	< 0.001		1.91 (1.46–2.50)	< 0.001
Q3	4.16 (3.26–5.30)	< 0.001	4.35 (3.38–5.60)	< 0.001		3.47 (2.62–4.59)	< 0.001
*P* for trend	< 0.001		< 0.001			< 0.001	
cuCTI-FI	1.11(1.10,1.13)	< 0.001	1.12(1.10,1.13)	< 0.001		1.10(1.09,1.12)	< 0.001
cuCTI3							
Q1	Ref		Ref			Ref	
Q2	2.43 (1.83–3.24)	< 0.001	2.62 (1.96–3.49)	< 0.001		2.30 (1.71–3.08)	< 0.001
Q3	5.70 (4.39–7.42)	< 0.001	6.53 (4.95–8.61)	< 0.001		5.27 (3.92–7.09)	< 0.001
*P* for trend	< 0.001		< 0.001			< 0.001	
Change in the CTI-FI							
Cluster 3	Ref		Ref			Ref	
Cluster 1	2.48 (2.02–3.03)	< 0.001	2.70 (2.19–3.32)		< 0.001	2.36 (1.89–2.94)	< 0.001
Cluster 2	5.23 (4.24–6.47)	< 0.001	5.88 (4.68–7.40)		< 0.001	4.89 (3.78–6.32)	< 0.001
*P* for trend	< 0.001		< 0.001			< 0.001	

Model 1: unadjusted for any covariates.

Model 2: adjusted for sex, age, marital status, and education level, location.

Model 3: adjusted for sex, age, marital status, education level, location, BMI, smoking status, drinking status, hypertension, cancer, HDL.

### Restricted cubic spline (RCS) analysis

**Fig. 3:**
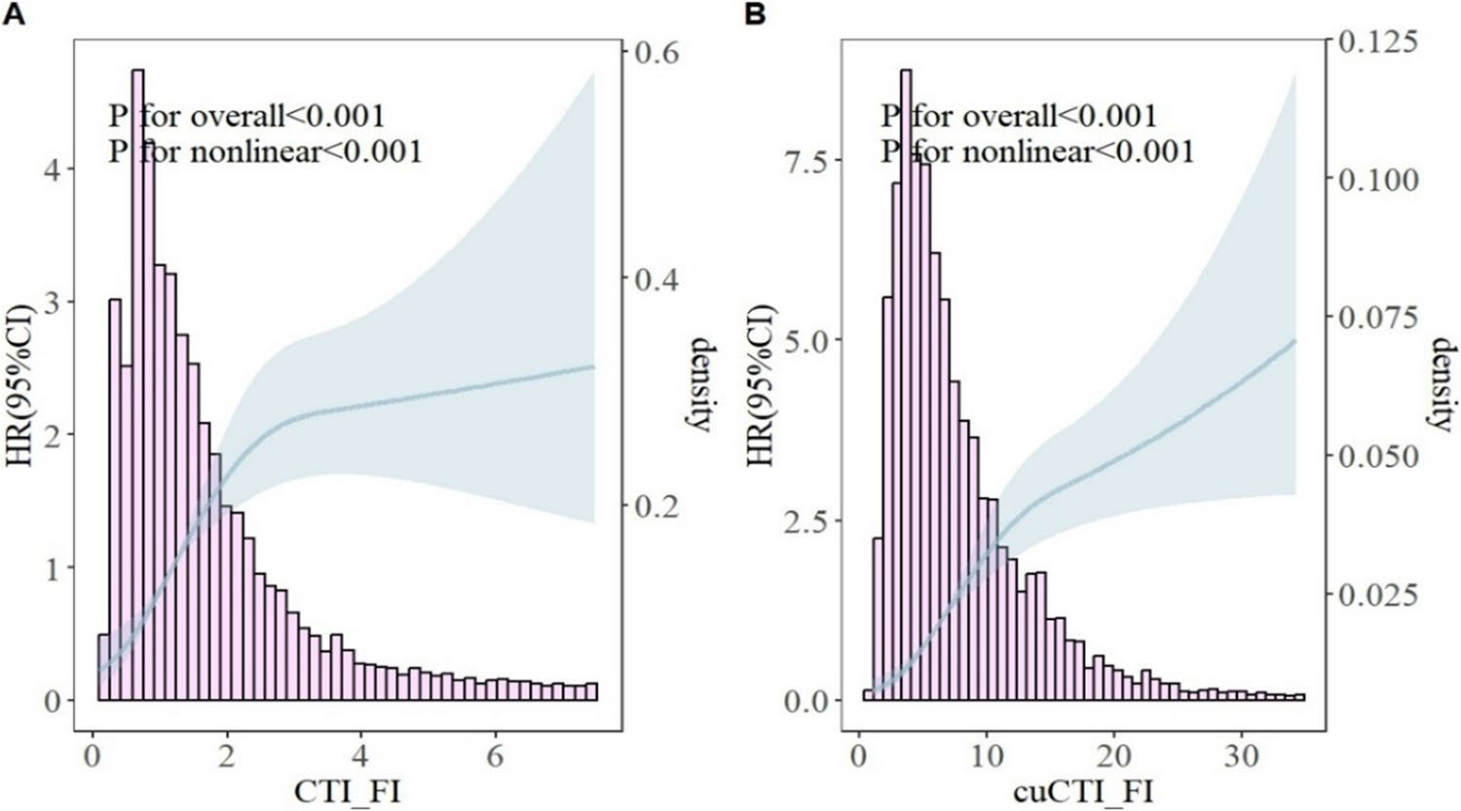
RCS curve between cuCTI-FI and CMM incidence. CTI-FI (**A**); cuCTI-FI (**B**)

Restricted cubic spline (RCS) analysis confirmed non-linear positive associations between both CTI-FI and cuCTI-FI and incident CMM (both P overall < 0.001, P non-linearity < 0.001; Fig. 3). For CTI-FI, the risk increased rapidly at lower levels before transitioning to a more gradual but sustained upward trend (Fig. 3A). In contrast, the HR for cuCTI-FI exhibited a steeper gradient at higher exposure levels, suggesting a cumulative risk-driving effect that intensifies over time (Fig. 3B). Sex-specific RCS curves are presented in Figure S2.

### K‒M survival and ROC curve analysis

**Fig. 4:**
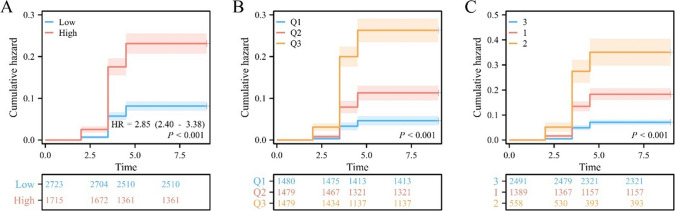
K‒M plot of CMM incidence according to CTI-FI (**A**), cuCTI-FI tertiles (**B**), and the CTI-FI cluster (**C**)

**Fig. 5:**
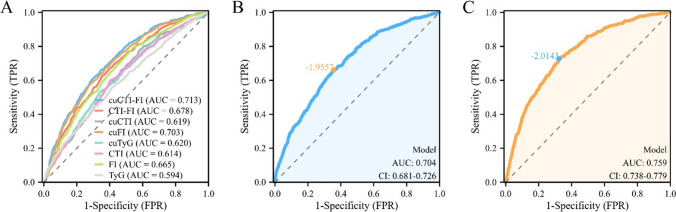
ROC curves of CMM incidence of TyG, CTI, FI, cuTyG, cuCTI, cuFI, CTI-FI and cuCTI-FI (**A**), China-par model (**B**), China-Par model with cuCTI-FI (**C**). C‑reactive protein–triglyceride–glucose index (CTI); triglyceride–glucose index (TyG); Frailty index (FI); Cumulative TyG (cuTyG), Cumulative CTI (cuCTI); Cumulative CTI-FI (cuCTI-FI)

Kaplan–Meier curves showed significant differences in CMM-free survival across CTI-FI tertiles, cuCTI-FI tertiles, and change pattern clusters (all log-rank P < 0.001; Fig. 4). In prediction analyses, cuCTI-FI showed a higher discriminative performance for incident CMM compared with cuFI and CTI-FI, with AUC values of 0.713, 0.703, and 0.678, respectively (Fig. 5). When cuCTI-FI was additionally incorporated into the China-PAR-based model, the AUC increased from 0.704 (95% CI: 0.682–0.726) to 0.760 (95% CI: 0.739–0.780).

The enhanced model showed improved performance, with lower Brier score (0.1007 vs. 0.1042) and mean absolute error (0.1995 vs. 0.2081), while maintaining good calibration (calibration slope ≈1.0; Table S8). Time-dependent ROC analysis showed higher AUCs for the enhanced model across different follow-up periods (Table S9), and decision curve analysis suggested greater net benefit across relevant threshold probabilities (Figure S3). These results suggest that incorporating cuCTI-FI may provide additional prognostic information beyond the China-PAR model.

### Subgroup analysis and interaction tests

**Fig. 6:**
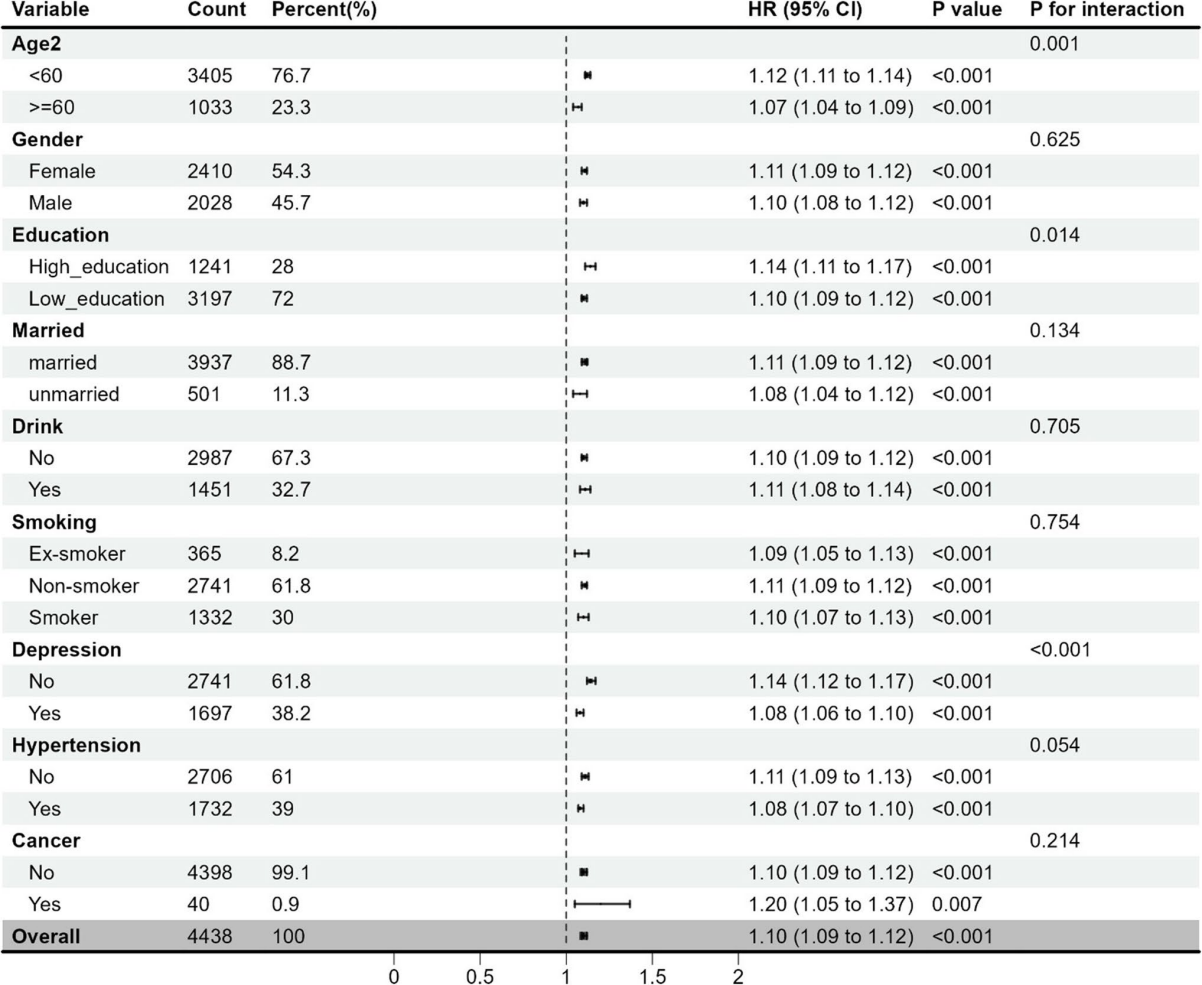
Subgroup and interaction analyses of the associations between cuCTI-FI and CMM risk

Exploratory subgroup analyses suggested that the association between cuCTI-FI and incident CMM was generally consistent across various strata (Fig. 6, Tables S5–S7). Significant statistical interactions were observed for age, education level, and depressive status (all P interaction < 0.05). The magnitude of association appeared to differ across these subgroups, with relatively higher HRs observed among participants aged < 60 years and those with higher education. However, these findings should be interpreted cautiously given the exploratory nature of subgroup analyses. No significant interactions were observed for sex, smoking status, or other baseline characteristics.

### Sensitivity analyses

Several sensitivity analyses were performed to assess the impact of missing data, baseline comorbidities, survey wave selection, and competing risk of death. The associations of CTI-FI and cuCTI-FI with incident CMM remained generally consistent after excluding participants with missing covariates, excluding participants with baseline hypertension or cancer, and excluding those from the 2020 follow-up wave. Fine–Gray competing risk models considering death as a competing event also yielded similar estimates (Table 3).

**Table 3:** Sensitivity analyses of the associations between CTI-FI/cuCTI-FI and incident cardiometabolic multimorbidity

	Model 1		Model 2		Model 3	
	HR(95%CI)	*P*_*adj*_	HR(95%CI)	*P*_*adj*_	HR(95%CI)	*P*_*adj*_
Exclude participants with missing data (N = 3985)						
CTI-FI	1.50(1.41–1.60)	< 0.001	1.51(1.42–1.61)	< 0.001	1.36(1.26–1.47)	< 0.001
CTI-FI3						
Q1	Ref		Ref		Ref	
Q2	2.13(1.62–2.79)	< 0.001	2.17(1.65–2.85)	< 0.001	1.89(1.43–2.49)	< 0.001
Q3	4.12(3.21–5.30)	< 0.001	4.32(3.32–5.62)	< 0.001	3.26(2.44–4.36)	< 0.001
*P* for trend	< 0.001		< 0.001		< 0.001	
cuCTI-FI	1.10(1.09–1.12)	< 0.001	1.11(1.09,1.12)	< 0.001	1.09(1.07,1.11)	< 0.001
cuCTI3						
Q1	Ref		Ref		Ref	
Q2	2.65(1.95–3.58)	< 0.001	2.86(2.11–3.88)	< 0.001	2.46(1.81–3.35)	< 0.001
Q3	6.13(4.64–8.10)	< 0.001	7.07(5.27–9.48)	< 0.001	5.52(4.03–7.56)	< 0.001
*P* for trend	< 0.001		< 0.001		< 0.001	
Exclude participants with hypertension or cancer (N = 2682)						
CTI-FI	1.51(1.38–1.66)		1.50(1.35–1.65)	< 0.001	1.45(1.29–1.63)	< 0.001
CTI-FI3						
Q1	Ref		Ref		Ref	
Q2	2.20(1.52–3.19)	< 0.001	2.23(1.53–3.24)	< 0.001	2.21(1.51–3.24)	< 0.001
Q3	4.03(2.84–5.71)	< 0.001	4.04(2.80–5.84)	< 0.001	4.06(2.67–6.16)	< 0.001
*P* for trend	< 0.001		< 0.001		< 0.001	
cuCTI-FI	1.13(1.10–1.16)	< 0.001	1.13(1.10–1.16)	< 0.001	1.13(1.10–1.17)	< 0.001
cuCTI3						
Q1	Ref		Ref		Ref	
Q2	2.55(1.72–3.82)	< 0.001	2.67(1.80–4.04)	< 0.001	2.68(1.78–4.08)	< 0.001
Q3	5.37(3.72–7.92)	< 0.001	5.73(3.86–8.69)	< 0.001	5.94(3.83–9.38)	< 0.001
*P* for trend	< 0.001		< 0.001		< 0.001	
Excluding participants from the 2020 follow-up wave (N = 4299)						
CTI-FI	1.53(1.43–1.63)	< 0.001	1.55(1.45–1.66)	< 0.001	1.39(1.28–1.51)	< 0.001
CTI-FI3						
Q1	Ref		Ref		Ref	
Q2	2.16(1.58–2.95)	< 0.001	2.24(1.64–3.06)	< 0.001	1.92(1.40–2.64)	< 0.001
Q3	4.66(3.51–6.19)	< 0.001	5.08(3.78–6.84)	< 0.001	3.75(2.71–5.19)	< 0.001
*P* for trend	< 0.001		< 0.001		< 0.001	
cuCTI-FI	1.13(1.11–1.15)	< 0.001	1.51(1.42–1.61)	< 0.001	1.13(1.10–1.15)	< 0.001
cuCTI3						
Q1	Ref		Ref		Ref	
Q2	2.50(1.78–3.56)	< 0.001	2.71(1.92–3.88)	< 0.001	2.30 (1.62–3.31)	< 0.001
Q3	6.94(5.10–9.63)	< 0.001	8.16(5.88–11.5)	< 0.001	6.26 (4.39–9.07)	< 0.001
*P* for trend	< 0.001		< 0.001		< 0.001	
Fine–Gray competing risk analyses						
CTI-FI	1.50(1.42–1.58)	< 0.001	1.50(1.42–1.59)	< 0.001	1.35(1.25–1.46)	< 0.001
CTI-FI3						
Q1	Ref		Ref		Ref	
Q2	2.09(1.62–2.70)	< 0.001	2.13(1.64–2.76)	< 0.001	1.87(1.43–2.43)	< 0.001
Q3	3.98(3.15–5.04)	< 0.001	4.14(3.22–5.32)	< 0.001	3.16(2.40–4.18)	< 0.001
*P* for trend	< 0.001		< 0.001		< 0.001	
cuCTI-FI	1.11(1.10–1.12)	< 0.001	1.11(1.10–1.13)	< 0.001	1.10(1.08–1.12)	< 0.001
cuCTI3						
Q1	Ref		Ref		Ref	
Q2	2.40(1.81–3.17)	< 0.001	2.56(1.92–3.41)	< 0.001	2.27 (1.70–3.03)	< 0.001
Q3	5.42(4.19–7.01)	< 0.001	6.13(4.66–8.08)	< 0.001	5.00 (3.74–6.68)	< 0.001
*P* for trend	< 0.001		< 0.001		< 0.001	

Model 1: unadjusted for any covariates.

Model 2: adjusted for sex, age, marital status, and education level, location.

Model 3: adjusted for sex, age, marital status, education level, location, BMI, smoking status, drinking status, hypertension, cancer, HDL. In the sensitivity analysis excluding participants with hypertension or cancer, these two variables were not included in Model 3 adjustment.

## Discussion

In this nationwide prospective cohort study of middle-aged and older Chinese adults, we constructed and evaluated the CTI-FI, a composite index integrating inflammatory status (CRP), metabolic dysfunction (TyG), and functional vulnerability (FI). We found that both baseline CTI-FI and cumulative CTI-FI exposure (cuCTI-FI) were positively associated with incident cardiometabolic multimorbidity (CMM), with higher levels associated with greater risk. In addition, incorporating cuCTI-FI into a China-PAR-based model was associated with improved discrimination and prediction performance metrics, suggesting that CTI-FI may provide additional information for identifying individuals with higher cardiometabolic vulnerability.

The development of CMM is likely influenced by multiple interconnected biological processes, including metabolic dysfunction, chronic inflammation, and declining physiological reserve [31]. Previous studies have investigated various composite indicators [32] that integrate metabolic abnormalities, obesity-related factors, or functional status to capture cardiometabolic risk [33, 34]. For example, indices such as TyG-WHtR [35] and TyG-FI [18] have been associated with cardiometabolic outcomes by reflecting metabolic dysfunction combined with anthropometric or functional characteristics. However, most existing indicators focus on limited biological domains. Recent longitudinal studies have also suggested that modified cardiometabolic indices incorporating multiple metabolic components [10, 36] and cumulative exposure indicators may better reflect long-term biological burden than single baseline measurements. In this context, CTI-FI incorporates inflammatory, metabolic [37, 38], and functional dimensions within a longitudinal framework, providing a complementary approach for characterizing cumulative cardiometabolic vulnerability.

Our findings further highlight the potential relevance of repeated measurements. Compared with baseline CTI-FI alone, cuCTI-FI, which incorporates changes over time, showed a stronger association with incident CMM. The clustering analysis identified different CTI-FI change patterns, with participants showing persistently higher or increasing CTI-FI levels exhibiting higher CMM incidence. However, these patterns should be interpreted as observed changes between available measurement waves rather than definitive biological trajectories. Future studies with more frequent measurements are needed to further clarify the dynamic evolution of cardiometabolic vulnerability.

The addition of cuCTI-FI to the China-PAR-based model resulted in improved discrimination, calibration-related performance, and decision-analytic measures. China-PAR was originally developed for cardiovascular disease risk assessment, and in the present study it was used as a reference model representing conventional cardiometabolic risk assessment. Therefore, the observed improvement suggests that cuCTI-FI may provide complementary information beyond traditional risk factors [39], although further validation in independent populations is required before clinical implementation.

In exploratory subgroup analyses, the association between cuCTI-FI and incident CMM appeared to vary across some participant characteristics, including age, education level, and depressive status. These findings may reflect differences in population characteristics or measurement patterns; however, they should be interpreted cautiously because subgroup analyses were exploratory and require confirmation in future studies. We therefore avoided drawing mechanistic conclusions from these observations.

From a practical perspective, CTI-FI is based on routinely available laboratory measurements and a frailty assessment, which may facilitate its application in epidemiological studies and community-based screening settings. Nevertheless, whether CTI-FI can improve clinical decision-making or population health management requires further evaluation through external validation and prospective implementation studies.

Several limitations should be acknowledged. First, although we adjusted for multiple potential confounders, residual confounding from unmeasured factors, such as dietary patterns and other lifestyle factors, cannot be completely excluded. Second, some cardiometabolic conditions were based on self-reported information, which may introduce misclassification. Third, CTI-FI is a composite indicator constructed from available inflammatory, metabolic, and functional measures; although it showed associations with CMM risk, the optimal weighting and biological interpretation of this index require further investigation. Fourth, the China-PAR model was originally developed for cardiovascular risk prediction rather than CMM, and the incremental predictive value of CTI-FI requires confirmation in external populations. Fifth, the clustering analysis was based on measurements from only two available waves; therefore, the identified patterns should be interpreted as observed changes rather than definitive trajectories. Finally, participants were community-dwelling middle-aged and older Chinese adults, limiting generalizability to other populations or clinical settings. Although multiple imputation was applied to address missing data, potential bias related to missingness cannot be fully eliminated.

## Conclusion

In conclusion, higher baseline and cumulative CTI-FI levels were associated with an increased risk of incident CMM among middle-aged and older Chinese adults. The incorporation of CTI-FI, particularly cumulative CTI-FI, provided additional prognostic information beyond conventional risk factors in this population. These findings suggest that integrating inflammatory, metabolic, and functional indicators may help characterize cardiometabolic vulnerability; however, further validation in independent populations is needed before its broader application.

## Supplementary Information

Below is the link to the electronic supplementary material.

Supplementary file1 (DOCX 450 KB)

## Data Availability

The data supporting the findings of this study are available on the CHARLS website (https://charls.pku.edu.cn/)

## Abbreviations

CMM: Cardiometabolic multimorbidity​
CTI: C-reactive protein–triglyceride–glucose index​
CTI-FI: C-reactive protein–triglyceride–glucose–frailty index​
cuCTI-FI: Cumulative C-reactive protein–triglyceride–glucose–frailty index​
CRP: C-reactive protein​
TyG: Triglyceride-glucose index​
FI: Frailty index​
CHARLS: China Health and Retirement Longitudinal Study​
AUC: Area under the receiver operating characteristic curve​
HR: Hazard ratio​
CI: Confidence interval
