# Supplementary material for: Long-term cumulative exposure and change patterns of CTI-FI predict the risk of cardiometabolic multimorbidity in middle-aged and older Chinese adults
Source: J Diabetes Metab Disord. 2026 Jul 24;25(2):207. doi: 10.1007/s40200-026-02026-y (PMC13400651; doi:10.1007/s40200-026-02026-y)
Supplement: Supplementary file 1 — Supplementary file1 (DOCX 450 KB) [file 40200_2026_2026_MOESM1_ESM.docx]

Supplementary table legend and figure legend

[**Table S1 The information of missing data** 2](#_Toc235093892)

[**Table S2 The results of multicollinearity assessment** 2](#_Toc235093893)

[**Table S3 Baseline characteristics according to baseline cuCTI-FI tertiles** 3](#_Toc235093894)

[**Table S4 Baseline characteristics according to CTI-FI cluster** 4](#_Toc235093895)

[**Table S5 The subgroup analysis results of cuCTI-FI tertiles** 6](#_Toc235093896)

[**Table S6 The Subgroup analysis results of CTI-FI** 7](#_Toc235093897)

[**Table S7 The subgroup analysis results of CTI-FI tertiles** 8](#_Toc235093898)

[**Table S8. Comparison of predictive performance between base and enhanced models** 10](#_Toc235093899)

[**Table S9. Time-dependent AUC (95% CI) at different follow-up years** 10](#_Toc235093900)

[**Table S10.** The 29 items used to construct the frailty index. 11](#_Toc235093901)

[**Figure S1. Determination of optimal cluster number and stability assessment.** 13](#_Toc235093902)

[**Figure S2 RCS curve between CTI-FI, cuCTI-FI and CMM incidence.** 13](#_Toc235093903)

[**Figure S3. Performance comparison of the China-par model and cuCTI-FI model for predicting CMM.** 14](#_Toc235093904)

**Table S1 The information of missing data**

| Variable | Missing_Count | Total_Sample | Missing_Rate |
| --- | --- | --- | --- |
| Depression | 238 | 4438 | 5.36 |
| DBP | 51 | 4438 | 1.15 |
| SBP | 48 | 4438 | 1.08 |
| Liver disease | 39 | 4438 | 0.88 |
| Kidney disease | 35 | 4438 | 0.79 |
| Hypertension | 29 | 4438 | 0.65 |
| BMI | 28 | 4438 | 0.63 |
| Cancer | 27 | 4438 | 0.61 |
| Height | 21 | 4438 | 0.47 |
| WHtR | 21 | 4438 | 0.47 |
| Lung disease | 21 | 4438 | 0.47 |
| Asthma | 20 | 4438 | 0.45 |
| Stomach | 18 | 4438 | 0.41 |
| Weight | 13 | 4438 | 0.29 |
| LDL | 10 | 4438 | 0.23 |

**Table S2 The results of multicollinearity assessment**

| Variable | GVIF | DF | GVIF_sqrt_DF |
| --- | --- | --- | --- |
| WHtR | 480.1716 | 1 | 21.913 |
| BMI | 465.7586 | 1 | 21.581 |
| TC | 16.49117 | 1 | 4.061 |
| LDL | 13.41459 | 1 | 3.663 |
| TG | 7.029929 | 1 | 2.651 |
| HDL | 3.94127 | 1 | 1.985 |
| Weight | 3.913634 | 1 | 1.978 |
| Waist | 3.772196 | 1 | 1.942 |
| Height | 2.689884 | 1 | 1.64 |
| SBP | 2.658911 | 1 | 1.631 |
| DBP | 2.567918 | 1 | 1.602 |
| TyG | 1.542569 | 1 | 1.242 |
| Glucose | 1.466283 | 1 | 1.211 |
| Age | 1.380844 | 1 | 1.175 |
| Diabetes | 1.365576 | 1 | 1.169 |
| Lung disease | 1.149232 | 1 | 1.072 |
| Asthma | 1.110994 | 1 | 1.054 |
| Stomach disease | 1.086803 | 1 | 1.042 |
| Arthritis | 1.08073 | 1 | 1.04 |
| Heart problem | 1.060883 | 1 | 1.03 |
| Kidney disease | 1.047243 | 1 | 1.023 |
| Memory disease | 1.036684 | 1 | 1.018 |
| Emotional disease | 1.030147 | 1 | 1.015 |
| Liver disease | 1.026334 | 1 | 1.013 |
| Stroke | 1.016656 | 1 | 1.008 |
| Cancer | 1.005473 | 1 | 1.003 |

**Table S3 Baseline characteristics according to baseline cuCTI-FI tertiles**

|  | level | Q1  (N=1480) | Q2  (N=1479) | Q3  (N=1479) | P value |
| --- | --- | --- | --- | --- | --- |
| Age (median [IQR]) |  | 56.0[50.0,62.0] | 58.0[52.0,64.0] | 61.0[56.0,68.0] | <0.001 |
| Age group (%) | <60 | 1254(84.7) | 1157(78.2) | 994(67.2) | <0.001 |
|  | >=60 | 226(15.3) | 322(21.8) | 485(32.8) |  |
| Gender (%) | Female | 640(43.2) | 770(52.1) | 1000(67.6) | <0.001 |
|  | Male | 840(56.8) | 709(47.9) | 479(32.4) |  |
| Education (%) | High education | 626(42.3) | 407(27.5) | 208(14.1) | <0.001 |
|  | Low education | 854(57.7) | 1072(72.5) | 1271(85.9) |  |
| Married (%) | Married | 1375(92.9) | 1325(89.6) | 1237(83.6) | <0.001 |
|  | Unmarried | 105(7.1) | 154(10.4) | 242(16.4) |  |
| Location (%) | City | 118(8.0) | 83(5.6) | 48(3.2) | <0.001 |
|  | Village | 1362(92.0) | 1396(94.4) | 1431(96.8) |  |
| Drink (%) | No | 867(58.6) | 977(66.1) | 1143(77.3) | <0.001 |
|  | Yes | 613(41.4) | 502(33.9) | 336(22.7) |  |
| Smoking (%) | Ex-smoker | 109(7.4) | 130(8.8) | 126(8.5) | <0.001 |
|  | Non-smoker | 822(55.5) | 888(60.0) | 1031(69.7) |  |
|  | Smoker | 549(37.1) | 461(31.2) | 322(21.8) |  |
| Hypertension (%) | No | 1093(74.3) | 888(60.3) | 707(48.2) | <0.001 |
|  | Yes | 378(25.7) | 584(39.7) | 759(51.8) |  |
| Heart problem(%) | No | 1407(95.1) | 1361(92.0) | 1238(83.7) | <0.001 |
|  | Yes | 73(4.9) | 118(8.0) | 241(16.3) |  |
| Diabetes (%) | No | 1435(97.4) | 1379(93.8) | 1352(91.7) | <0.001 |
|  | Yes | 39(2.6) | 91(6.2) | 122(8.3) |  |
| Lung disease (%) | No | 1435(97.2) | 1314(89.4) | 1222(83.0) | <0.001 |
|  | Yes | 41(2.8) | 155(10.6) | 250(17.0) |  |
| Cancer (%) | No | 1472(99.7) | 1452(99.0) | 1448(98.6) | 0.005 |
|  | Yes | 4(0.3) | 15(1.0) | 20(1.4) |  |
| Liver disease (%) | No | 1442(97.9) | 1400(95.7) | 1381(94.4) | <0.001 |
|  | Yes | 31(2.1) | 63(4.3) | 82(5.6) |  |
| Stroke (%) | No | 1472(99.7) | 1463(99.5) | 1433(97.4) | <0.001 |
|  | Yes | 4(0.3) | 8(0.5) | 38(2.6) |  |
| Kidney disease (%) | No | 1420(96.4) | 1389(94.6) | 1331(91.0) | <0.001 |
|  | Yes | 53(3.6) | 79(5.4) | 131(9.0) |  |
| Stomach disease (%) | No | 1261(85.4) | 1120(76.0) | 1007(68.6) | <0.001 |
|  | Yes | 216(14.6) | 354(24.0) | 462(31.4) |  |
| Emotional disease (%) | No | 1474(99.6) | 1462(98.9) | 1443(97.6) | <0.001 |
|  | Yes | 6(0.4) | 17(1.1) | 36(2.4) |  |
| Memory disease (%) | No | 1470(99.3) | 1469(99.3) | 1457(98.5) | 0.031 |
|  | Yes | 10(0.7) | 10(0.7) | 22(1.5) |  |
| Arthritis (%) | No | 1265(85.5) | 942(63.7) | 689(46.6) | <0.001 |
|  | Yes | 215(14.5) | 537(36.3) | 790(53.4) |  |
| Asthma (%) | No | 1453(98.6) | 1419(96.3) | 1381(93.8) | <0.001 |
|  | Yes | 20(1.4) | 54(3.7) | 91(6.2) |  |
| Incident CMM during follow-up (%) | No | 1413(95.5) | 1321(89.3) | 1137(76.9) | <0.001 |
|  | Yes | 67(4.5) | 158(10.7) | 342(23.1) |  |
| BMI (median [IQR]) |  | 22.8[20.9,25.2] | 23.1[20.8,25.6] | 23.4[20.9,26.4] | 0.001 |
| Waist (median [IQR]) |  | 83.2[77.1,90.0] | 84.3[78.0,91.4] | 86.1[78.8,93.2] | <0.001 |
| SBP (median [IQR]) |  | 123.7[113.3,136.3] | 127.0[114.3,141.7] | 130.0[115.7,147.3] | <0.001 |
| DBP (median [IQR]) |  | 73.7[66.7,81.7] | 75.0[67.0,83.1] | 75.7[67.7,84.0] | 0.001 |
| Glucose (median [IQR]) |  | 5.6[5.2,6.1] | 5.7[5.3,6.2] | 5.7[5.3,6.2] | <0.001 |
| TG (median [IQR]) |  | 1.1[0.8,1.5] | 1.1[0.8,1.6] | 1.2[0.9,1.8] | <0.001 |
| TC (median [IQR]) |  | 4.9[4.3,5.5] | 4.9[4.3,5.6] | 5.0[4.4,5.7] | 0.004 |
| HDL (median [IQR]) |  | 1.3[1.1,1.6] | 1.3[1.0,1.6] | 1.3[1.0,1.5] | 0.015 |
| LDL (median [IQR]) |  | 3.0[2.4,3.5] | 3.0[2.5,3.5] | 3.0[2.4,3.6] | 0.707 |

**Table S4 Baseline characteristics according to CTI-FI cluster**

|  | level | Cluster 1  (N=1389) | Cluster 2  (N=558) | Cluster 3  (N=2491) | P  value |
| --- | --- | --- | --- | --- | --- |
| Age (median [IQR]) |  | 60.0[54.0,66.0] | 63.0[58.0,69.0] | 57.0[51.0,63.0] | <0.001 |
| Age group (%) | <60 | 1014(73.0) | 337(60.4) | 2054(82.5) | <0.001 |
|  | >=60 | 375(27.0) | 221(39.6) | 437(17.5) |  |
| Gender (%) | Female | 875(63.0) | 384(68.8) | 1151(46.2) | <0.001 |
|  | Male | 514(37.0) | 174(31.2) | 1340(53.8) |  |
| Education (%) | High education | 255(18.4) | 59(10.6) | 927(37.2) | <0.001 |
|  | Low education | 1134(81.6) | 499(89.4) | 1564(62.8) |  |
| Married (%) | Married | 1190(85.7) | 454(81.4) | 2293(92.1) | <0.001 |
|  | Unmarried | 199(14.3) | 104(18.6) | 198(7.9) |  |
| Location (%) | City | 50(3.6) | 20(3.6) | 179(7.2) | <0.001 |
|  | Village | 1339(96.4) | 538(96.4) | 2312(92.8) |  |
| Drink (%) | No | 1012(72.9) | 444(79.6) | 1531(61.5) | <0.001 |
|  | Yes | 377(27.1) | 114(20.4) | 960(38.5) |  |
| Smoking (%) | Ex-smoker | 113(8.1) | 50(9.0) | 202(8.1) | <0.001 |
|  | Non-smoker | 924(66.5) | 385(69.0) | 1432(57.5) |  |
|  | Smoker | 352(25.3) | 123(22.0) | 857(34.4) |  |
| Hypertension (%) | No | 726(52.6) | 240(43.3) | 1722(69.6) | <0.001 |
|  | Yes | 654(47.4) | 314(56.7) | 753(30.4) |  |
| Heart problem (%) | No | 1213(87.3) | 451(80.8) | 2342(94.0) | <0.001 |
|  | Yes | 176(12.7) | 107(19.2) | 149(6.0) |  |
| Diabetes (%) | No | 1277(92.5) | 505(90.7) | 2384(96.1) | <0.001 |
|  | Yes | 104(7.5) | 52(9.3) | 96(3.9) |  |
| Lung disease (%) | No | 1166(84.3) | 452(81.6) | 2353(94.9) | <0.001 |
|  | Yes | 217(15.7) | 102(18.4) | 127(5.1) |  |
| Cancer (%) | No | 1357(98.6) | 549(98.7) | 2466(99.5) | 0.015 |
|  | Yes | 19(1.4) | 7(1.3) | 13(0.5) |  |
| Liver disease (%) | No | 1309(95.1) | 509(92.9) | 2405(97.2) | <0.001 |
|  | Yes | 68(4.9) | 39(7.1) | 69(2.8) |  |
| Stroke (%) | No | 1354(98.0) | 536(96.8) | 2478(99.8) | <0.001 |
|  | Yes | 27(2.0) | 18(3.2) | 5(0.2) |  |
| Kidney disease (%) | No | 1271(92.3) | 491(89.6) | 2378(96.0) | <0.001 |
|  | Yes | 106(7.7) | 57(10.4) | 100(4.0) |  |
| Stomach disease (%) | No | 970(70.2) | 373(67.5) | 2045(82.3) | <0.001 |
|  | Yes | 411(29.8) | 180(32.5) | 441(17.7) |  |
| Emotional disease (%) | No | 1364(98.2) | 541(97.0) | 2474(99.3) | <0.001 |
|  | Yes | 25(1.8) | 17(3.0) | 17(0.7) |  |
| Memory disease (%) | No | 1375(99.0) | 545(97.7) | 2476(99.4) | 0.001 |
|  | Yes | 14(1.0) | 13(2.3) | 15(0.6) |  |
| Arthritis (%) | No | 670(48.2) | 245(43.9) | 1981(79.5) | <0.001 |
|  | Yes | 719(51.8) | 313(56.1) | 510(20.5) |  |
| Asthma (%) | No | 1303(94.2) | 523(94.1) | 2427(97.9) | <0.001 |
|  | Yes | 80(5.8) | 33(5.9) | 52(2.1) |  |
| Incident CMM during follow-up (%) | No | 1157(83.3) | 393(70.4) | 2321(93.2) | <0.001 |
|  | Yes | 232(16.7) | 165(29.6) | 170(6.8) |  |
| BMI (median [IQR]) |  | 23.3[20.8,26.0] | 23.3[20.9,26.4] | 23.0[20.9,25.4] | 0.052 |
| Waist (median [IQR]) |  | 85.1[78.0,92.4] | 87.0[79.2,94.0] | 84.0[77.5,90.6] | <0.001 |
| SBP (median [IQR]) |  | 128.7[115.0,144.3] | 132.3[118.2,150.0] | 124.7[113.7,138.0] | <0.001 |
| DBP (median [IQR]) |  | 74.7[67.0,83.7] | 76.0[68.8,84.3] | 74.3[67.0,82.3] | 0.001 |
| Glucose (median [IQR]) |  | 5.7[5.3,6.2] | 5.8[5.3,6.4] | 5.6[5.2,6.1] | <0.001 |
| TG (median [IQR]) |  | 1.2[0.9,1.7] | 1.2[0.9,1.8] | 1.1[0.8,1.6] | <0.001 |
| TC (median [IQR]) |  | 5.0[4.4,5.6] | 5.0[4.4,5.6] | 4.9[4.3,5.5] | 0.019 |
| HDL (median [IQR]) |  | 1.3[1.1,1.6] | 1.2[1.0,1.5] | 1.3[1.1,1.6] | 0.008 |
| LDL (median [IQR]) |  | 3.0[2.4,3.6] | 3.0[2.4,3.6] | 3.0[2.4,3.5] | 0.686 |

**Table S5 The subgroup analysis results of cuCTI-FI tertiles**

| Variable | Count | Percent (%) | Levels | HR (95% CI) | P value | P for interaction |
| --- | --- | --- | --- | --- | --- | --- |
| Age group |  |  |  |  |  | 0.028 |
| <60 | 3405 | 76.7 | Q1 |  |  |  |
|  |  |  | Q2 | 2.63 (1.90 to 3.64) | <0.001 |  |
|  |  |  | Q3 | 6.87 (5.09 to 9.26) | <0.001 |  |
| >=60 | 1033 | 23.3 | Q1 |  |  |  |
|  |  |  | Q2 | 1.71 (0.94 to 3.12) | 0.081 |  |
|  |  |  | Q3 | 3.08 (1.78 to 5.31) | <0.001 |  |
| Gender |  |  |  |  |  | 0.11 |
| Female | 2410 | 54.3 | Q1 |  |  |  |
|  |  |  | Q2 | 1.94 (1.27 to 2.96) | 0.002 |  |
|  |  |  | Q3 | 5.47 (3.76 to 7.95) | <0.001 |  |
| Male | 2028 | 45.7 | Q1 |  |  |  |
|  |  |  | Q2 | 2.98 (2.02 to 4.40) | <0.001 |  |
|  |  |  | Q3 | 5.67 (3.88 to 8.28) | <0.001 |  |
| Education |  |  |  |  |  | 0.224 |
| High education | 1241 | 28 | Q1 |  |  |  |
|  |  |  | Q2 | 3.32 (2.16 to 5.10) | <0.001 |  |
|  |  |  | Q3 | 6.66 (4.31 to 10.28) | <0.001 |  |
| Low education | 3197 | 72 | Q1 |  |  |  |
|  |  |  | Q2 | 2.14 (1.46 to 3.15) | <0.001 |  |
|  |  |  | Q3 | 5.87 (4.15 to 8.30) | <0.001 |  |
| Married |  |  |  |  |  | 0.432 |
| Married | 3937 | 88.7 | Q1 |  |  |  |
|  |  |  | Q2 | 2.57 (1.90 to 3.46) | <0.001 |  |
|  |  |  | Q3 | 5.85 (4.44 to 7.71) | <0.001 |  |
| Unmarried | 501 | 11.3 | Q1 |  |  |  |
|  |  |  | Q2 | 1.37 (0.52 to 3.66) | 0.526 |  |
|  |  |  | Q3 | 4.38 (1.89 to 10.18) | 0.001 |  |
| Drink |  |  |  |  |  | 0.292 |
| No | 2987 | 67.3 | Q1 |  |  |  |
|  |  |  | Q2 | 2.86 (1.96 to 4.19) | <0.001 |  |
|  |  |  | Q3 | 6.62 (4.66 to 9.42) | <0.001 |  |
| Yes | 1451 | 32.7 | Q1 |  |  |  |
|  |  |  | Q2 | 1.91 (1.23 to 2.99) | 0.004 |  |
|  |  |  | Q3 | 4.35 (2.86 to 6.61) | <0.001 |  |
| Smoking |  |  |  |  |  | 0.875 |
| Ex-smoker | 365 | 8.2 | Q1 |  |  |  |
|  |  |  | Q2 | 2.63 (1.12 to 6.18) | 0.027 |  |
|  |  |  | Q3 | 5.43 (2.42 to 12.15) | <0.001 |  |
| Non-smoker | 2741 | 61.8 | Q1 |  |  |  |
|  |  |  | Q2 | 2.36 (1.59 to 3.49) | <0.001 |  |
|  |  |  | Q3 | 6.02 (4.22 to 8.59) | <0.001 |  |
| Smoker | 1332 | 30 | Q1 |  |  |  |
|  |  |  | Q2 | 2.47 (1.53 to 3.99) | <0.001 |  |
|  |  |  | Q3 | 5.06 (3.20 to 8.01) | <0.001 |  |
| Depression |  |  |  |  |  | 0.175 |
| No | 2741 | 61.8 | Q1 |  |  |  |
|  |  |  | Q2 | 2.60 (1.90 to 3.55) | <0.001 |  |
|  |  |  | Q3 | 5.31 (3.89 to 7.26) | <0.001 |  |
| Yes | 1697 | 38.2 | Q1 |  |  |  |
|  |  |  | Q2 | 2.70 (1.16 to 6.32) | 0.022 |  |
|  |  |  | Q3 | 7.92 (3.52 to 17.82) | <0.001 |  |
| Hypertension |  |  |  |  |  | 0.695 |
| No | 2706 | 61 | Q1 |  |  |  |
|  |  |  | Q2 | 2.46 (1.67 to 3.62) | <0.001 |  |
|  |  |  | Q3 | 5.02 (3.50 to 7.21) | <0.001 |  |
| Yes | 1732 | 39 | Q1 |  |  |  |
|  |  |  | Q2 | 1.96 (1.28 to 3.01) | 0.002 |  |
|  |  |  | Q3 | 4.54 (3.06 to 6.72) | <0.001 |  |
| Cancer |  |  |  |  |  | 0.248 |
| No | 4398 | 99.1 | Q1 |  |  |  |
|  |  |  | Q2 | 2.45 (1.84 to 3.27) | <0.001 |  |
|  |  |  | Q3 | 5.70 (4.39 to 7.41) | <0.001 |  |
| Yes | 40 | 0.9 | Q1 |  |  |  |
|  |  |  | Q2 | 1.00 (0.00 to Inf) | 1 |  |
|  |  |  | Q3 | 646923400.95 (0.00 to Inf) | 0.999 |  |
| Overall | 4438 | 100 | Q1 |  |  |  |
|  |  |  | Q2 | 2.43 (1.83 to 3.24) | <0.001 |  |
|  |  |  | Q3 | 5.70 (4.39 to 7.41) | <0.001 |  |

**Table S6 The Subgroup analysis results of CTI-FI**

| Variable | Count | Percent (%) | HR (95% CI) | P value | P for interaction |
| --- | --- | --- | --- | --- | --- |
| Age group |  |  |  |  | 0.001 |
| <60 | 3405 | 76.7 | 1.58 (1.48 to 1.68) | <0.001 |  |
| >=60 | 1033 | 23.3 | 1.24 (1.09 to 1.40) | 0.001 |  |
| Gender |  |  |  |  | 0.939 |
| Female | 2410 | 54.3 | 1.47 (1.37 to 1.58) | <0.001 |  |
| Male | 2028 | 45.7 | 1.48 (1.33 to 1.64) | <0.001 |  |
| Education |  |  |  |  | 0.031 |
| High education | 1241 | 28 | 1.69 (1.50 to 1.91) | <0.001 |  |
| Low education | 3197 | 72 | 1.46 (1.36 to 1.56) | <0.001 |  |
| Married |  |  |  |  | 0.024 |
| Married | 3937 | 88.7 | 1.52 (1.43 to 1.62) | <0.001 |  |
| Unmarried | 501 | 11.3 | 1.23 (1.03 to 1.46) | 0.025 |  |
| Drink |  |  |  |  | 0.87 |
| No | 2987 | 67.3 | 1.46 (1.37 to 1.56) | <0.001 |  |
| Yes | 1451 | 32.7 | 1.49 (1.30 to 1.69) | <0.001 |  |
| Smoking |  |  |  |  | 0.765 |
| Ex-smoker | 365 | 8.2 | 1.41 (1.21 to 1.65) | <0.001 |  |
| Non-smoker | 2741 | 61.8 | 1.50 (1.39 to 1.61) | <0.001 |  |
| Smoker | 1332 | 30 | 1.44 (1.26 to 1.64) | <0.001 |  |
| Depression |  |  |  |  | <0.001 |
| No | 2741 | 61.8 | 1.73 (1.55 to 1.93) | <0.001 |  |
| Yes | 1697 | 38.2 | 1.35 (1.25 to 1.47) | <0.001 |  |
| Hypertension |  |  |  |  | 0.077 |
| No | 2706 | 61 | 1.52 (1.38 to 1.66) | <0.001 |  |
| Yes | 1732 | 39 | 1.35 (1.25 to 1.46) | <0.001 |  |
| Cancer |  |  |  |  | 0.276 |
| No | 4398 | 99.1 | 1.48 (1.40 to 1.57) | <0.001 |  |
| Yes | 40 | 0.9 | 2.29 (1.04 to 5.06) | 0.04 |  |
| Overall | 4438 | 100 | 1.48 (1.40 to 1.57) | <0.001 |  |

**Table S7 The subgroup analysis results of CTI-FI tertiles**

| Variable | Count | Percent (%) | Levels | HR (95% CI) | P value | P for interaction |
| --- | --- | --- | --- | --- | --- | --- |
| Age group |  |  |  |  |  | 0.059 |
| <60 | 3405 | 76.7 | Q1 |  |  |  |
|  |  |  | Q2 | 2.43 (1.80 to 3.27) | <0.001 |  |
|  |  |  | Q3 | 4.85 (3.67 to 6.42) | <0.001 |  |
| >=60 | 1033 | 23.3 | Q1 |  |  |  |
|  |  |  | Q2 | 1.22 (0.70 to 2.13) | 0.483 |  |
|  |  |  | Q3 | 2.43 (1.50 to 3.94) | <0.001 |  |
| Gender |  |  |  |  |  | 0.02 |
| Female | 2410 | 54.3 | Q1 |  |  |  |
|  |  |  | Q2 | 1.91 (1.30 to 2.82) | 0.001 |  |
|  |  |  | Q3 | 4.66 (3.29 to 6.60) | <0.001 |  |
| Male | 2028 | 45.7 | Q1 |  |  |  |
|  |  |  | Q2 | 2.35 (1.65 to 3.36) | <0.001 |  |
|  |  |  | Q3 | 3.34 (2.35 to 4.76) | <0.001 |  |
| Education |  |  |  |  |  | 0.16 |
| High education | 1241 | 28 | Q1 |  |  |  |
|  |  |  | Q2 | 3.01 (1.95 to 4.63) | <0.001 |  |
|  |  |  | Q3 | 5.44 (3.52 to 8.39) | <0.001 |  |
| Low education | 3197 | 72 | Q1 |  |  |  |
|  |  |  | Q2 | 1.78 (1.28 to 2.48) | 0.001 |  |
|  |  |  | Q3 | 3.83 (2.84 to 5.16) | <0.001 |  |
| Married |  |  |  |  |  | 0.443 |
| Married | 3937 | 88.7 | Q1 |  |  |  |
|  |  |  | Q2 | 2.15 (1.63 to 2.83) | <0.001 |  |
|  |  |  | Q3 | 4.36 (3.37 to 5.63) | <0.001 |  |
| Unmarried | 501 | 11.3 | Q1 |  |  |  |
|  |  |  | Q2 | 1.74 (0.77 to 3.92) | 0.184 |  |
|  |  |  | Q3 | 2.75 (1.29 to 5.83) | 0.009 |  |
| Drink |  |  |  |  |  | 0.198 |
| No | 2987 | 67.3 | Q1 |  |  |  |
|  |  |  | Q2 | 2.32 (1.66 to 3.24) | <0.001 |  |
|  |  |  | Q3 | 4.71 (3.46 to 6.41) | <0.001 |  |
| Yes | 1451 | 32.7 | Q1 |  |  |  |
|  |  |  | Q2 | 1.81 (1.18 to 2.77) | 0.006 |  |
|  |  |  | Q3 | 2.98 (1.97 to 4.51) | <0.001 |  |
| Smoking |  |  |  |  |  | 0.16 |
| Ex-smoker | 365 | 8.2 | Q1 |  |  |  |
|  |  |  | Q2 | 2.28 (1.02 to 5.13) | 0.045 |  |
|  |  |  | Q3 | 4.22 (1.96 to 9.08) | <0.001 |  |
| Non-smoker | 2741 | 61.8 | Q1 |  |  |  |
|  |  |  | Q2 | 1.84 (1.30 to 2.62) | 0.001 |  |
|  |  |  | Q3 | 4.33 (3.17 to 5.91) | <0.001 |  |
| Smoker | 1332 | 30 | Q1 |  |  |  |
|  |  |  | Q2 | 2.56 (1.62 to 4.05) | <0.001 |  |
|  |  |  | Q3 | 3.38 (2.14 to 5.34) | <0.001 |  |
| Depression |  |  |  |  |  | 0.845 |
| No | 2741 | 61.8 | Q1 |  |  |  |
|  |  |  | Q2 | 2.16 (1.61 to 2.89) | <0.001 |  |
|  |  |  | Q3 | 4.09 (3.02 to 5.53) | <0.001 |  |
| Yes | 1697 | 38.2 | Q1 |  |  |  |
|  |  |  | Q2 | 2.28 (1.04 to 5.01) | 0.04 |  |
|  |  |  | Q3 | 4.79 (2.26 to 10.16) | <0.001 |  |
| Hypertension |  |  |  |  |  | 0.516 |
| No | 2706 | 61 | Q1 |  |  |  |
|  |  |  | Q2 | 2.19 (1.51 to 3.17) | <0.001 |  |
|  |  |  | Q3 | 4.04 (2.85 to 5.72) | <0.001 |  |
| Yes | 1732 | 39 | Q1 |  |  |  |
|  |  |  | Q2 | 1.65 (1.14 to 2.40) | 0.009 |  |
|  |  |  | Q3 | 3.03 (2.15 to 4.28) | <0.001 |  |
| Cancer |  |  |  |  |  | 0.355 |
| No | 4398 | 99.1 | Q1 |  |  |  |
|  |  |  | Q2 | 2.13 (1.64 to 2.77) | <0.001 |  |
|  |  |  | Q3 | 4.17 (3.27 to 5.31) | <0.001 |  |
| Yes | 40 | 0.9 | Q1 |  |  |  |
|  |  |  | Q2 | 1.00 (0.00 to Inf) | 1 |  |
|  |  |  | Q3 | 408181451.54 (0.00 to Inf) | 0.999 |  |
| Overall | 4438 | 100 | Q1 |  |  |  |
|  |  |  | Q2 | 2.12 (1.63 to 2.75) | <0.001 |  |
|  |  |  | Q3 | 4.16 (3.26 to 5.30) | <0.001 |  |

# **Table S8. Comparison of predictive performance between base and enhanced models**

| **Metric** | **China-par model** | **cuCTI-FI model** |
| --- | --- | --- |
| AUC (95% CI) | 0.704 (0.682-0.726) | 0.760 (0.739-0.780) |
| Brier Score | 0.1042 | 0.1007 |
| Calibration Slope (95% CI) | 1.000 (0.877-1.125) | 1.000 (0.899-1.103) |
| MAE | 0.2081 | 0.1995 |
| Net Benefit at 20% | 0.0139 | 0.0287 |
| NRI (95% CI) | - | 0.5125 (0.4269-0.5930) |
| IDI (95% CI) | - | 0.0385 (0.0302-0.0466) |

# **Table S9. Time-dependent AUC (95% CI) at different follow-up years**

| **Time(year)** | **China-par model AUC** | **cuCTI-FI model AUC** |
| --- | --- | --- |
| 3 | 0.790 (0.731-0.848) | 0.856 (0.815-0.898) |
| 5 | 0.790 (0.731-0.848) | 0.856 (0.815-0.898) |
| 8 | 0.703 (0.681-0.725) | 0.757 (0.737-0.778) |

# **Table S10.** The 29 items used to construct the frailty index.

| **Individuals components** | **Description of the items** | **Criteria** |
| --- | --- | --- |
| **Self-Reported Diseases** | Physician diagnosed hypertension | Yes = 1, No = 0 |
|  | Physician diagnosed cancer | Yes = 1, No = 0 |
|  | Physician diagnosed arthritis | Yes = 1, No = 0 |
|  | Physician diagnosed chronic lung disease | Yes = 1, No = 0 |
|  | Physician diagnosed asthma | Yes = 1, No = 0 |
|  | Physician diagnosed any emotional, nervous, or psychiatric problems | Yes = 1, No = 0 |
|  | Physician diagnosed memory-related disease | Yes = 1, No = 0 |
| **Self-Reported Vision and Hearing Issues** | Vision problems | Yes = 1, No = 0 |
|  | Hearing problems | Yes = 1, No = 0 |
| **Self-Reported General and Mental Health Assessment** | Self-reported general health status | Poor or fair = 1, excellent, very good, or good = 0 |
|  | Depression: CESD-10 questionnaire | CESD-10 >10 =1, ≤10 =0 |
|  | Cognition: (memory test score + orientation test score) **/** 14 | Continuous, ranging from 0 to 1 |
| **Activities of Daily Living (ADLs)** | Difficulty with dressing | Yes = 1, No = 0 |
|  | Difficulty with bathing or showering | Yes = 1, No = 0 |
|  | Difficulty with eating | Yes = 1, No = 0 |
|  | Difficulty with getting in and out of bed | Yes = 1, No = 0 |
|  | Difficulty with using the toilet | Yes = 1, No = 0 |
| **Instrumental Activities of Daily Living (IADLs)** | Difficulty with managing money | Yes = 1, No = 0 |
|  | Difficulty with taking medications | Yes = 1, No = 0 |
|  | Difficulty with shopping for groceries | Yes = 1, No = 0 |
|  | Difficulty with preparing meals | Yes = 1, No = 0 |
|  | Difficulty with doing housework | Yes = 1, No = 0 |
| **Mobility Status** | Mobility: difficulty with walking 100 yards | Yes = 1, No = 0 |
|  | Mobility: difficulty with getting up from a chair after sitting for long periods | Yes = 1, No = 0 |
|  | Mobility: difficulty with climbing several flights of stairs without resting | Yes = 1, No = 0 |
|  | Mobility: difficulty with lifting or carrying weights over 10 pounds/jins | Yes = 1, No = 0 |
|  | Mobility: difficulty with picking up a coin from the table | Yes = 1, No = 0 |
|  | Mobility: difficulty with stooping, kneeling, or crouching | Yes = 1, No = 0 |
|  | Mobility: difficulty with reaching arms above shoulder level | Yes = 1, No = 0 |


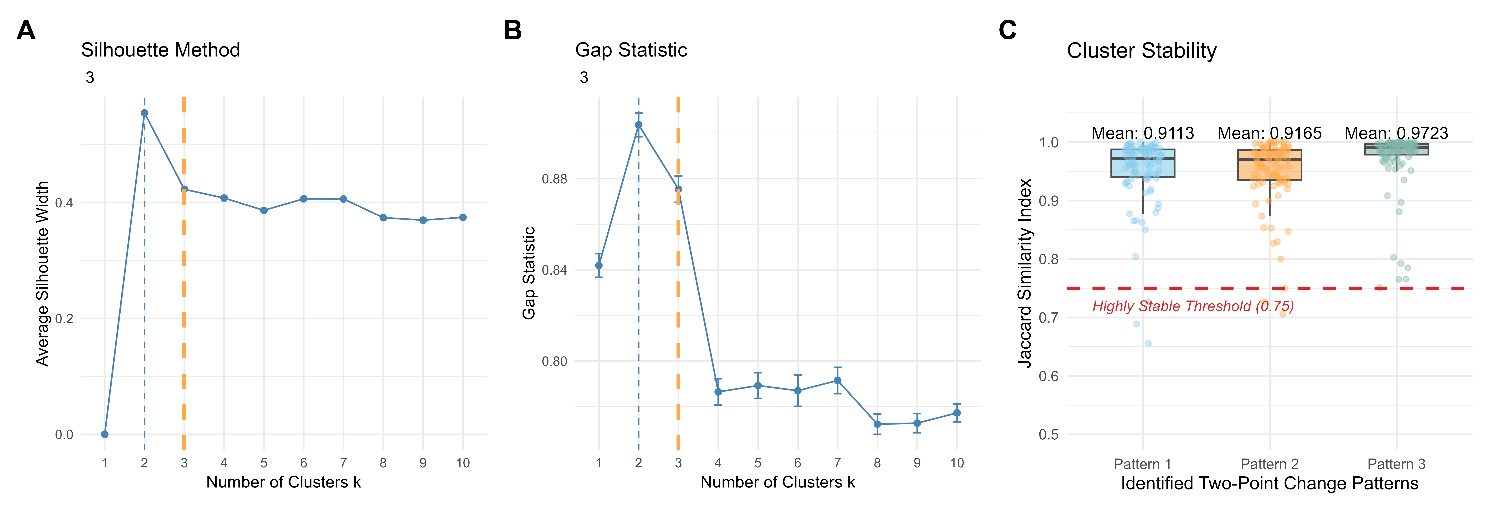


# **Figure S1. Determination of optimal cluster number and stability assessment.**

1. Silhouette analysis for measuring cluster cohesion and separation. (B) Gap statistic plot for estimating the optimal number of clusters. (C) Stability assessment of the final 3-cluster solution across repeated sampling.


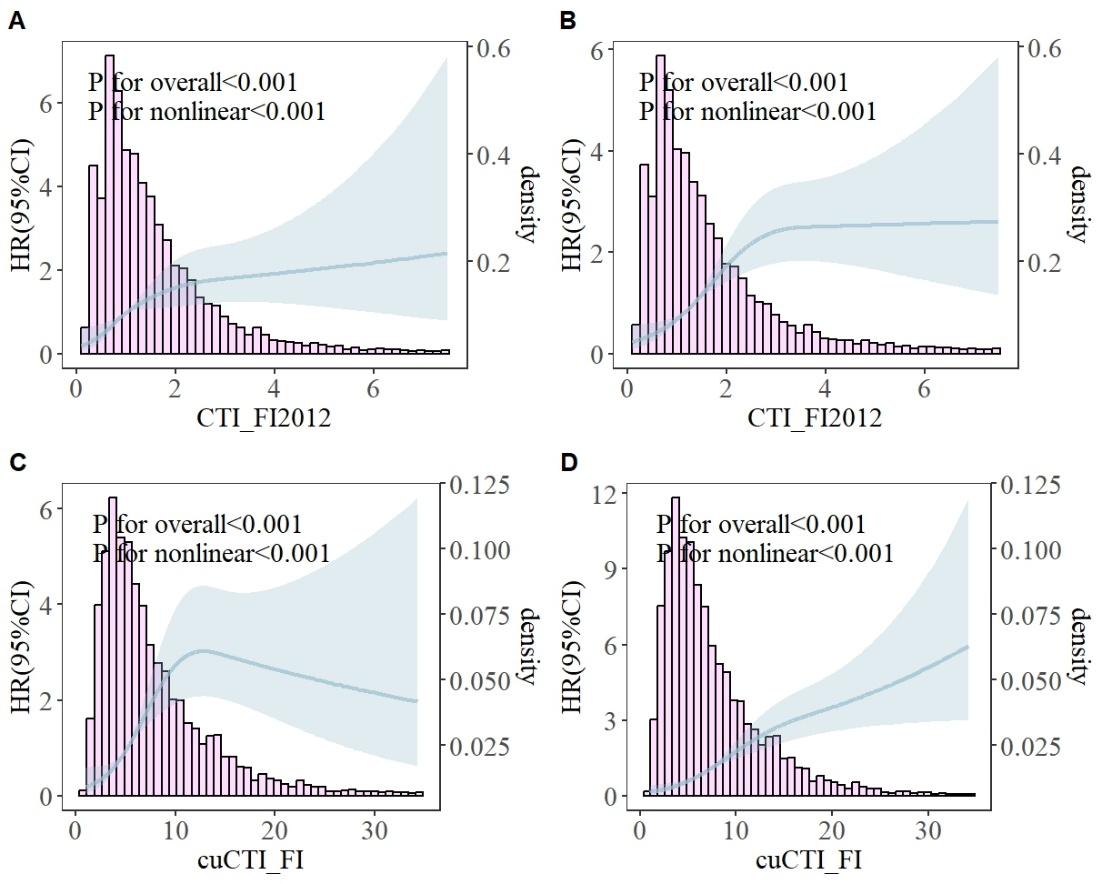


# **Figure S2 RCS curve between CTI-FI, cuCTI-FI and CMM incidence.**

1. CTI-FI in male; (B) CTI-FI in female; (C) cuCTI-FI in male; (D) cuCTI-FI in female.

**
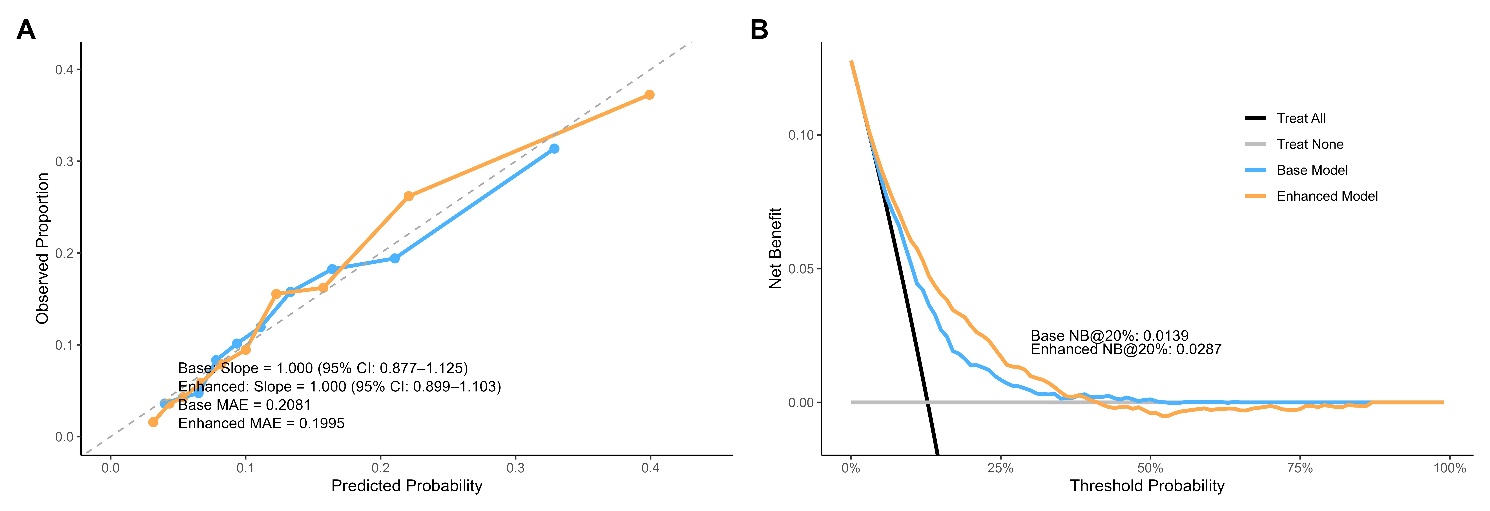
**

# **Figure S3. Performance comparison of the China-par model and cuCTI-FI model for predicting CMM.**

(A) Calibration curves showing the agreement between predicted and observed risks. (B) Decision curve analysis (DCA) comparing the clinical net benefit at a 20% risk threshold.
